# ^18^F-FDG PET/CT predicts acute exacerbation in idiopathic pulmonary fibrosis after thoracic surgery

**DOI:** 10.1186/s12890-021-01659-4

**Published:** 2021-09-16

**Authors:** Hee-Young Yoon, Suk Hyun Lee, Sejin Ha, Jin-Sook Ryu, Jin Woo Song

**Affiliations:** 1 Department of Pulmonary and Critical Care Medicine, Asan Medical Centre, University of Ulsan College of Medicine, 88 Olympic-Ro 43-Gil, Songpa-Gu, Seoul, 05505 Republic of Korea; 2 Department of Nuclear Medicine, Asan Medical Centre, University of Ulsan College of Medicine, 88 Olympic-Ro 43-Gil, Songpa-Gu, Seoul, 05505 Republic of Korea; 3 Division of Nuclear Medicine, Department of Radiology, Kangnam Sacred Heart Hospital, Hallym University College of Medicine, Singil-ro, Yeongdeungpo-gu, Seoul, 07441 Republic of Korea

**Keywords:** Idiopathic pulmonary fibrosis, Positron emission tomography/computed tomography, Acute exacerbation, Post-operative, Prediction

## Abstract

**Background:**

Acute exacerbation (AE) is the most lethal postoperative complication in idiopathic pulmonary fibrosis (IPF); however, prediction before surgery is difficult. We investigated the role of ^18^F-fluorodeoxyglucose positron emission tomography/computed tomography (^18^F-FDG PET/CT) in predicting postoperative AE in IPF.

**Method:**

Clinical data of 48 IPF patients who underwent 18F-FDG PET/CT before thoracic surgery were retrospectively analyzed. Mean and maximal standardized uptake values (SUV_mean_ and SUV_max_, respectively) were measured in the fibrotic area. Additionally, adjusted values-SUV ratio (SUVR, defined as SUV_max_-to-liver SUV_mean_ ratio), tissue fraction-corrected SUV_mean_ (SUV_meanTF_), and SUVR (SUVR_TF_)-were calculated.

**Results:**

The mean age of the subjects was 67.8 years and 91.7% were male. After thoracic surgery, 21 (43.8%) patients experienced postoperative complications including prolonged air leakage (29.2%), death (14.6%), and AE (12.5%) within 30 days. Patients who experienced AE showed higher SUV_max_, SUVR, SUV_meanTF_, and SUVR_TF_ than those who did not, but other clinical parameters were not different between patients with and without AE. The SUV parameters did not differ for other complications. The SUVR (odds ratio [OR] 29.262; *P* = 0.030), SUV_meanTF_ (OR 3.709; *P* = 0.041) and SUVR_TF_ (OR 20.592; *P* = 0.017) were significant predicting factors for postoperative AE following a multivariate logistic regression analysis. On receiver operating characteristics curve analysis, SUVR_TF_ had the largest area under the curve (0.806, *P* = 0.007) for predicting postoperative AE among SUV parameters.

**Conclusions:**

Our findings suggest that ^18^F-FDG PET/CT may be useful in predicting postoperative AE in IPF patients and among SUVs, SUVR_TF_ is the best parameter for predicting postoperative AE in IPF patients.

**Supplementary Information:**

The online version contains supplementary material available at 10.1186/s12890-021-01659-4.

## Introduction

Idiopathic pulmonary fibrosis (IPF) is a disorder characterized by chronic progressive pulmonary fibrosis of unknown etiology [1], but shows variable course, including acute exacerbation (AE). AE of IPF could be provoked by viral infection, aspiration, and mechanical stress such as that from thoracic surgery. After thoracic surgery, IPF patients may experience more frequent postoperative complications than non-IPF patients [2–6]. AE occurs in 3–25% of IPF patients after thoracic surgery and is the most lethal postoperative complication, with a mortality rate between 7 and 23% [2, 7–9]. Therefore, it is important to identify the population at risk for postoperative AE among IPF patients before surgery. Previous studies reported several risk factors for postoperative AE, including low lung function, poor performance status, high composite physiologic index (CPI), and high lactate dehydrogenase (LDH) levels, in IPF patients [5, 7, 10–12]. However, in another study involving 56 IPF patients, no association between clinical parameters (lung function, levels of surfactant protein-D [SP-D] and Krebs von den Lungen-6 [KL-6], and operation type and time) and postoperative AE was observed [6]. Due to these conflicting results, predictors for postoperative AE in IPF are not well defined.

^18^F-fluorodeoxyglucose positron emission tomography with computed tomography (^18^F-FDG PET/CT) can assess the metabolic activity of lung tissue by detecting increased FDG uptake [13]. Fibrotic lung parenchyma shows an increased uptake of FDG due to increased numbers of erythrocytes and inflammatory cells with glucose transporter-1 expression resulting from neovascularization [14]. Previous studies reported that the standardized uptake value (SUV), a semi-quantitative index for FDG uptake in PET/CT, was associated with lung function, levels of C-reactive protein [CRP], LDH, SP-D, and KL-6, and clinical outcomes (decline in lung function, transplant-free survival, and death) in IPF patients [15–18]. These results suggest that ^18^F-FDG PET/CT could provide additional information on disease activity and prognosis in IPF patients before thoracic surgery. Therefore, we aimed to investigate the usefulness of ^18^F-FDG PET/CT in predicting postoperative complications, including AE, in IPF patients.

## Materials and methods

### Subjects

**Fig. 1:**
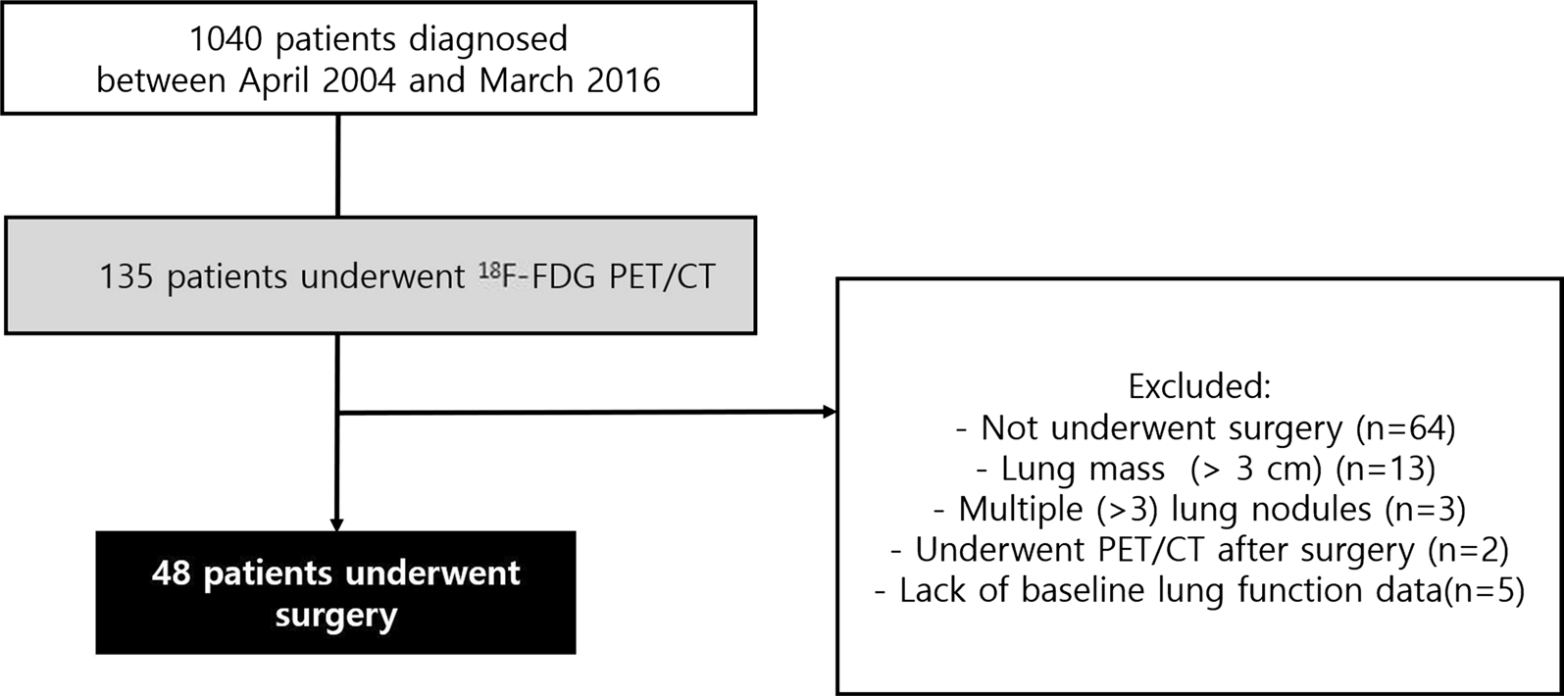
Flowchart of patient selection. IPF, idiopathic pulmonary fibrosis; ^18^F-FDG, ^18^F-fluorodeoxyglucose; PET/CT, positron emission tomography with computed tomography;

Between April 2004 and March 2016, 1040 IPF patients were diagnosed at Asan Medical Center, Seoul, South Korea and 135 patients who underwent ^18^F-FDG PET/CT were screened for this study. Among them, 87 patients were excluded because they had not undergone surgery (n = 64), had factors that could affect measurement of SUV in fibrotic area including lung masses (> 3 cm, n = 13), multiple (> 3) lung nodules (n = 3), and undergoing PET/CT after surgery (n = 2), and had no baseline lung function data (n = 5). Finally, 48 IPF patients (biopsy proven cases = 26) who underwent ^18^F-FDG PET/CT before thoracic surgery for lung nodule were enrolled in the study (Fig. 1). All patients were confirmed with IPF according to the diagnostic criteria of the American Thoracic Society (ATS)/European Respiratory Society/Japanese Respiratory Society/Latin American Thoracic Association statement [1]. This study was approved by the Asan Medical Center Institutional Review Board (2017–0057), and the need to obtain informed consent was waived due to the retrospective nature of the study.

### Clinical data

The clinical and survival data at the time of PET/CT were retrospectively obtained from medical records, telephone interviews, and/or the National Health Insurance of Korea. Spirometry, diffusing capacity of the lung for carbon monoxide (DLco), and total lung capacity (TLC) by plethysmography were measured according to recommendations [19–21]. The 6-min walk test (6MWT) was performed according to ATS guidelines [22]. All methods were performed in accordance with the relevant guidelines and regulations. The gender-age-physiology (GAP) index was calculated using the GAP model [23].

Postoperative complications were defined as the occurrence of the following events within 30 days after thoracic surgery: (1) AE of IPF; (2) prolonged air leakage via chest tube more than five days after thoracotomy; (3) any other event requiring treatment and extending hospitalization period; and (4) death. Based on an international working group report, we defined AE of IPF as an acute respiratory deterioration characterized by evidence of new widespread alveolar abnormality [24]. The Charlson comorbidity index (CCI) was used to evaluate the impact on of comorbidities on acute exacerbation [25].

### PET/CT imaging protocol and analysis

^18^F-FDG PET/CT and its imaging analysis were performed according to a previously documented protocol [26]. All patients underwent ^18^F-FDG PET/CT following fasting for at least six hours and blood glucose levels remained below 8.33 mmol/L (150 mg/dL) before PET/CT. PET/CT was conducted within 50–70 min after the injection of 5.18–7.4 MBq/kg (0.14–0.2 mCi/kg) of ^18^F-FDG. The following scanners were used for image acquisition: Biograph Sensation 16 (Siemens, Knoxville, TN, USA), Discovery STe 8 (GE Healthcare, Milwaukee, WI, USA), Biograph TruePoint 40 (Siemens), Discovery 690 Elite (GE Healthcare), Discovery 690 (GE Healthcare), and Discovery 710 (GE Healthcare). Depending on the PET/CT scanner used, three-dimensional PET images were obtained from the base of the skull to the mid-thigh with 5–8 beds for 2–3 min each. An iterative algorithm with attenuation correction in CT images was used to reconstruct PET images. The SUV, a common semi-quantification method for measuring FDG uptake in ^18^F-FDG PET/CT [27], was measured in the fibrosis area (red circle in Additional file 1: Fig. S1) by a certified physician (S.H.L., 8 years of experience in nuclear medicine). The SUV of the lung tissue was calculated according to the following equation: SUV = mean regional FDG activity (Bq/mL)/(injected activity [Bq]/body weight [g]).

The maximum SUV (SUV_max_) and mean SUV (SUV_mean_, the mean of SUVs measured by drawing a circle with a diameter of 1 cm centered on the measurement point of SUV_max_, Additional file 1: Fig. S1-D) of fibrotic areas were obtained except for a nodule on chest CT. To minimize the confounding effects of inhomogeneous density of fibrotic lung, different resolutions of various PET/CT machines, and measurement methods, adjusted values were calculated based on the SUV_max_ and SUV_mean_. To compensate for the SUV differences between individuals, the SUV ratio (SUVR) was calculated by dividing the SUV_max_ of the fibrotic area by the SUV_mean_ of the liver (measured by drawing a 3 cm-sized circle in the right hepatic lobe) [28], and to compensate for the adjustment for air component in the lung tissue [29], the tissue fraction corrected mean SUV (SUV_meanTF_) and SUVR (SUVR_TF_, defined as SUV_meanTF_ -to-liver SUV_mean_ ratio) were calculated.

### Statistical analysis

All values are presented as the mean ± standard deviation for continuous variables or as number (percentages) for categorical variables. For comparison between two groups, Mann–Whitney U test for continuous variables and Fisher's exact test for categorical variables were used. Logistic regression analysis was performed to determine risk factors for postoperative complications. In the multivariate analysis, the variables with a *P* value of < 0.05 in the unadjusted analysis or those considered to be clinically significant in previous studies [30, 31] such as GAP (gender, age, and physiology) index was included. Receiver operating characteristic (ROC) curve analysis was used to assess the performance of SUVs in predicting the development of postoperative complications. The discrimination power for postoperative complications was expressed using Area under the ROC curve (AUC). All statistical analyses were performed using SPSS 24.0 (IBM Corp.). A *P* < 0.05 was considered statistically significant (two-tailed).

## Results

### Study population

The mean age of all patients was 67.8 years, 91.7% of patients were males, and 93.7% were ever-smokers. Baseline lung function and exercise capacity were relatively preserved, and most patients had GAP stage I (68.8%) or II (29.2%) (Table 1). The median time from PET/CT to surgery was 11.5 days (interquartile range [IQR], 5.8–19.8 days). All patients underwent thoracic surgery due to suspected or confirmed malignant nodules, and 43 (89.6%) were finally diagnosed with malignant neoplasms. The most common type of surgery was lobectomy (56.3%), followed by wedge resection (35.4%) and segmentectomy (8.3%).

**Table 1:** Comparison of baseline characteristics between IPF patients with and without postoperative complications

Characteristics	Total	Complications	No-complications	*P* value
Patient No	48	21	27	
Age, year	67.8 ± 7.1	70.5 ± 6.2	65.7 ± 7.1	0.019
Male	44 (91.7)	21 (100.0)	23 (85.2)	0.121
Ever-smokers	45 (93.7)	21 (100.0)	24 (88.9)	0.246
FVC, % predicted	82.0 ± 12.7	82.8 ± 13.8	81.5 ± 12.0	0.732
DLco, % predicted	64.6 ± 18.0	65.2 ± 16.5	64.1 ± 19.4	0.838
TLC, % predicted	79.1 ± 10.5	79.7 ± 11.8	78.6 ± 9.6	0.719
6MWD, meter	457.4 ± 100.1	471.6 ± 104.0	446.0 ± 97.4	0.400
6MWT, resting SpO_2_, %	96.6 ± 1.3	96.3 ± 1.5	96.9 ± 1.1	0.139
6MWT, lowest SpO_2_, %	91.3 ± 5.0	90.4 ± 4.8	92.0 ± 5.2	0.307
GAP index	3.1 ± 1.2	3.3 ± 1.1	2.9 ± 1.3	0.308
CCI	4.8 ± 1.5	5.2 ± 1.3	4.5 ± 1.5	0.095
SUV parameters				
SUV_max_	2.1 ± 0.6	2.2 ± 0.6	1.9 ± 0.6	0.128
SUV_mean_	1.7 ± 0.4	1.8 ± 0.5	1.6 ± 0.4	0.137
SUVR	0.9 ± 0.3	1.0 ± 0.3	0.9 ± 0.2	0.227
SUV_meanTF_	2.5 ± 0.7	2.7 ± 0.7	2.4 ± 0.6	0.059
SUVR_TF_	1.3 ± 0.4	1.5 ± 0.5	1.3 ± 0.3	0.096

Data are presented as either mean ± standard deviation or number (percentage), unless otherwise indicated

IPF, idiopathic pulmonary fibrosis; FVC, forced vital capacity; DLco, diffusing capacity of the lung for carbon monoxide; TLC, total lung capacity; 6MWD, six-minute walk distance; 6MWT, six-minute walk test; SpO_2_, saturation of oxygen; GAP: gender, age, and physiology; CCI, Charlson comorbidity index; SUV, standardized uptake value; SUV_max_, maximum standardized uptake value; SUV_mean_, mean standardized uptake value; SUVR, standardized uptake value ratio; SUV_meanTF_, tissue fraction-corrected mean standardized uptake; SUVR_TF_, tissue fraction-corrected standardized uptake value ratio

Twenty-one (43.8%) patients experienced postoperative complications within 30 days after surgery. The most common complication was prolonged air leakage (29.2%), followed by death (14.6%), and AE (12.5%). Total 7 (14.6%) patients experienced multiple postoperative complications and patients who underwent lobectomy showed a significant tendency for the high rate of multiple complications. Prolonged air leakage, AE, and death were more frequent among patients who underwent lobectomy compared to those who did not, but there was no statistical significance (Additional file 1: Table S1).

### Baseline clinical characteristics and SUVs

Patients who experienced postoperative complications were older than those who did not. However, there were no significant differences in terms of gender, smoking history, lung function, exercise capacity, GAP index, and comorbidities between the two groups (Table 1). There were no differences in baseline clinical characteristics between patients with and without AE (Additional file 1: Table S2).

**Fig. 2:**
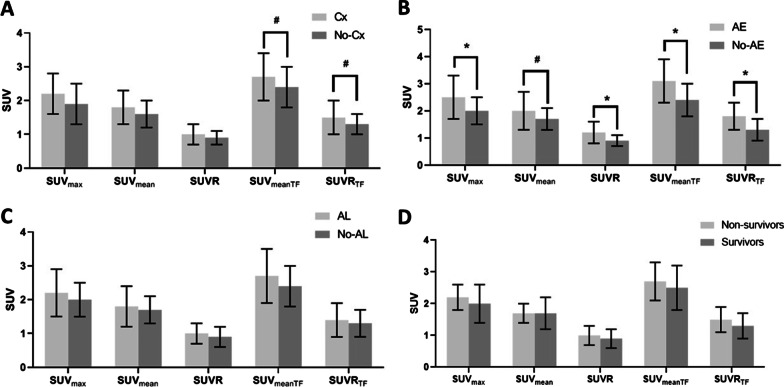
Comparison of baseline SUVs between IPF patients with and without postoperative complications. **A** All complications. **B** Acute exacerbation. **C** Prolonged air leakage. **D** Death. Each bar represents the mean and standard deviation. *p< 0.05, ^#^*p* < 0.1. SUV, maximum standardized uptake value; IPF, idiopathic pulmonary fibrosis; Cx, complications; SUV_max_, maximum standardized uptake value; SUV_mean_, mean standardized uptake value; SUVR, standardized uptake value ratio; SUV_meanTF_, tissue fraction-corrected mean standardized uptake; SUVR_TF_, tissue fraction-corrected standardized uptake value ratio; AE, acute exacerbation; AL, air leakage

In terms of SUV parameters, there were no significant differences between patients with and without postoperative complications (Table 1, Fig. 2A). However, all SUV parameters, except the SUV_mean_, in patients with postoperative AE were significantly higher than those in patients without postoperative AE (Fig. 2B, Additional file 1: Table S2). The SUV parameters were not significantly different between patients with and without prolonged air leakage (Fig. 2C) and between survivors and non-survivors (Fig. 2D).

### Predictors of postoperative acute exacerbation

Age was only significantly associated with the development of postoperative complications during univariate analysis (Additional file 1: Table S3). In the univariate logistic analysis, the SUVR, and SUVR_TF_ were significantly associated with postoperative AE, while the SUV_max_ and resting saturation of oxygen (SpO_2_) showed marginal significance (Table 2). In the multivariate analysis including GAP index, the SUVR (odds ratio [OR] 29.262; 95% confidence interval [CI] 1.379–621.125; *P* = 0.030), SUV_meanTF_ (OR 3.709; 95% CI 1.052–13.080; *P* = 0.041), and SUVR_TF_ (OR 20.592; 95% CI 1.725–245.841; *P* = 0.017) were independent risk factors for postoperative AE, while other SUVs showed marginal significance in predicting postoperative AE (Table 3).

**Table 2:** Risk factors for postoperative acute exacerbation in IPF patients assessed using univariate logistic regression analysis

Characteristics	Odds ratio (95% confidence interval)	*P* value
Age	1.100 (0.921–1.314)	0.292
Type of surgery*	5.000 (0.537–46.528)	0.157
FVC	0.979 (0.912–1.052)	0.569
DLco	1.024 (0.972–1.078)	0.379
TLC	0.967 (0.888–1.053)	0.435
6MWD	1.006 (0.993–1.019)	0.398
6MWT, resting SpO_2_	0.468 (0.246–1.006)	0.052
6MWT, lowest SpO_2_	0.950 (0.803–1.124)	0.550
GAP index	0.814 (0.392–1.694)	0.583
CCI	1.094 (0.606–1.974)	0.765
SUV_max_	3.699 (0.855–15.999)	0.080
SUV_mean_	4.432 (0.714–27.496)	0.110
SUVR	33.044 (1.312–832.424)	0.034
SUV_meanTF_	2.564 (0.707–9.294)	0.152
SUVR_TF_	12.329 (1.060–143.431)	0.045

IPF, idiopathic pulmonary fibrosis; FVC, forced vital capacity; DLco, diffusing capacity of the lung for carbon monoxide; TLC, total lung capacity; 6MWD, six-minute walk distance; 6MWT, six-minute walk test; SpO_2_, saturation of oxygen; GAP: gender, age, and physiology; CCI, Charlson comorbidity index; SUV, standardized uptake value; SUV_max_, maximum standardized uptake value; SUV_mean_, mean standardized uptake value; SUVR, standardized uptake value ratio; SUV_meanTF_, tissue fraction-corrected mean standardized uptake; SUVR_TF_, tissue fraction-corrected standardized. * lobectomy vs. others (wedge resection or segmentectomy)

**Table 3:** Risk factors for postoperative acute exacerbation in IPF patients assessed using multivariate logistic regression analysis

Characteristics*	Odds ratio (95% confidence interval)	*P* value
SUV_max_	3.680 (0.913–14.840)	0.067
SUV_mean_	4.660 (0.806–26.941)	0.086
SUVR	29.262 (1.379–621.125)	0.030
SUV_meanTF_	3.709 (1.052–13.080)	0.041
SUVR_TF_	20.592 (1.725–245.841)	0.017

IPF, idiopathic pulmonary fibrosis; SUV, standardized uptake value; SUV_max_, maximum standardized uptake value; SUV_mean_, mean standardized uptake value; SUVR, standardized uptake value ratio; SUV_meanTF_, tissue fraction-corrected mean standardized uptake; SUVR_TF_, tissue fraction-corrected standardized

*Each variable was adjusted by GAP index

### Performance of SUV parameters

**Fig. 3:**
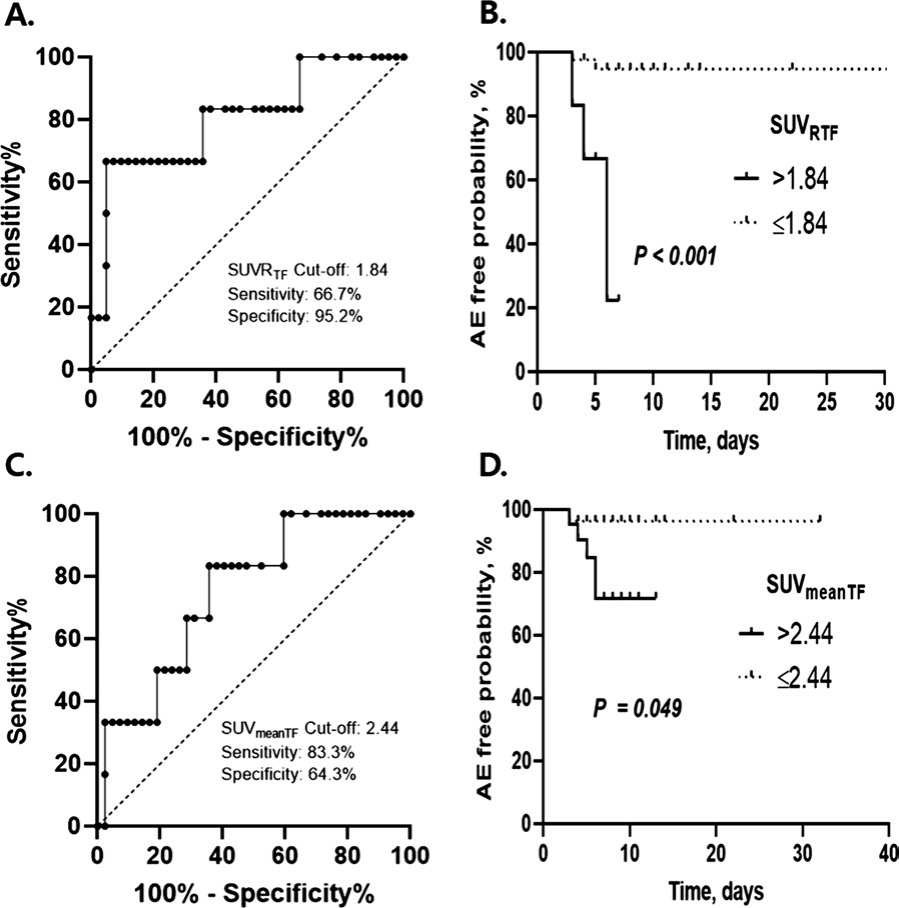
The receiver operating characteristic and Kaplan Meier’s survival curves of SUVs for acute exacerbation in patients with IPF. **A** Receiver operating characteristic curve of SUVR_TF_ for AE. **B** Comparison of AE free survival curves between patients with values above and below the best cut-off value of the SUVR_TF_. **C** Receiver operating characteristic curve of SUV_meanTF_ for predicting AE. **D** Comparison of AE free survival curves between patients with values above and below the best cut-off value of the SUV_meanTF_. Differences between the two groups were assessed using the log rank test. IPF, idiopathic pulmonary fibrosis; AE, acute exacerbation; SUV_meanTF_, tissue fraction-corrected mean standardized uptake; SUVR_TF_, tissue fraction-corrected standardized uptake value ratio

In the ROC curve analysis, the SUVR_TF_ showed significance in predicting postoperative AE (AUC = 0.806; 95% CI 0.666–0.905; *P* = 0.007) and the best cut-off value was 1.84 (sensitivity: 66.7%, specificity: 95.2%, positive predictive value [PPV]: 66.7%, negative predictive value [NPV]: 75.2%) (Fig. 3A). Patients with high SUVR_TF_ showed lower 30-day AE free survival rate than those with low SUVR_TF_ (33.3% [> 1.84] vs. 95.2% [≤ 1.84]; *P* < 0.001; Fig. 3B). The SUV_meanTF_ also significantly predicted postoperative AE with best cut-off value of 2.44 (AUC = 0.754; 95% CI 0.608–0.867; *P* = 0.010; sensitivity: 66.7% specificity: 71.4%, PPV: 23.8%, NPV: 76.3%; Fig. 3C), and patients with high SUV_meanTF_ had lower 30-day AE free survival rate than those with low SUV_meanTF_ (76.2% [> 2.44] vs. 96.3% [≤ 2.44]; *P* = 0.049; Fig. 3D). There was no difference in discrimination power between SUVR_TF_ and SUV_meanTF_ (*P* = 0.339). However, baseline SUV_mean_ (AUC = 0.655; 95% CI 0.396–0.913; *P* = 0.224), SUVR (AUC = 0.702; 95% CI 0.452–0.953; *P* = 0.112) and SUV_max_ (AUC = 0.700; 95% CI 0.485–0.915; *P* = 0.115) could not predict postoperative AE in IPF patients.

## Discussion

In this study, increased SUV was associated with postoperative AE in IPF patients. Among SUV parameters, the SUVR and SUVR_TF_ were independent predictors for postoperative AE in IPF patients, and the SUVR_TF_ was the best parameter for predicting postoperative AE in IPF patients.

Although lung function of the subjects was relatively preserved in our study, 43.8% of subjects experienced postoperative complications including AE (12.5%), which were similar to those of previous studies [6, 32, 33]. Saito et al. reported that 10.7% and 40.7% of IPF patients (n = 28, mean vital capacity: 87.1%) with stage IA non-small cell lung cancer (NSCLC) developed postoperative AE and complications, respectively [32]. Watanabe et al., in IPF patients with lung cancer (n = 56, vital capacity: 103.8%, DLco: 61.4%), also reported that 7.1% experienced postoperative AE [6]. Moreover, Otsuka et al., in IPF patients with lung cancer (n = 9), reported that 44.4% experienced AE after thoracic surgery although lung function of the subjects was not impaired (mean vital capacity: 89% predicted, DLco: 73% predicted) [33]. These results suggest that occurrence of AE is not uncommon after thoracic surgery even in IPF patients with relatively preserved lung function.

Patient demographics and baseline lung function were not associated with postoperative AE in IPF patients in our study. Our findings are consistent with those of previous studies [6, 34]. Watanabe et al. reported that clinical parameters (vital capacity, DLco, white blood cell count, CRP, LDH, SP-D. KL-6, operation time and type, and histopathologic cancer type) were not different between IPF patients suffering from lung cancer with (n = 4) and without (n = 52) postoperative AE after lung resection [6]. However, other studies showed different results [5, 7, 10–12, 35]. Sato et al. reported that in 1763 patients with interstitial lung disease (ILD, including 1235 IPF) who underwent thoracic surgery for lung cancer, the male gender, history of previous acute exacerbation, preoperative steroid use, serum KL-6 levels, low vital capacity, usual interstitial pneumonia pattern on chest CT scan, and type of surgery were independent predicting factors for postoperative AE [35]. Kumar et al., in 22 IPF patients with NSCLC, also showed that postoperative acute respiratory distress syndrome (ARDS) was associated with low baseline DLco and high CPI [7]. In addition, Kusibe et al. reported that baseline FVC in 33 IPF patients with lung cancer was lower in patients who developed acute lung injury or ARDS (n = 9) after lung resection (74.0 vs. 103.7% predicted, P < 0.001) compared to those without acute lung injury or ARDS [5]. These inconsistent results suggest that clinical variables might be insufficient to predict the occurrence of postoperative AE in IPF patients.

In our study, SUV parameters such as SUVR, SUV_meanTF_, and SUVR_TF_ were only significant predictors for postoperative AE in IPF patients. No study has demonstrated the role of PET/CT in predicting postoperative complications in IPF patients. However, some studies suggested that FDG uptake was associated with severity and prognosis in IPF patients [15–18]. Lee et al. reported significant correlation between SUV and baseline lung function (FVC: r = -0.6, *P* = 0.024; DLco: r = -0.7, *P* = 0.001) in 8 IPF patients [15]. Low baseline lung function was reported to be associated with postoperative AE in IPF patients [5, 7, 10]. Nobashi et al. reported that SUV parameters (SUV_max_, SUV_mean_, SUV_meanTF_) in 90 patients with ILD (including 24 IPF) were correlated with baseline CRP and LDH, which are risk factors for postoperative AE in ILD patients [10]. These findings support the role of SUV parameters in predicting postoperative AE in IPF. Justet et al. also reported that in 27 IPF patients, lung volume adjusted SUV metrics, were significantly associated with disease progression including AE, using the multivariate Cox analysis adjusted by age, FVC, and DLco [18]. Therefore, these results suggest that patients with high SUV levels in the fibrotic area need measures to prevent acute exacerbation such as preoperative antifibrotic treatment, and careful observation after surgery.

In our study, SUVR_TF_, which was corrected for both individual variation and air component of lung tissue, was an independent predictor and was the best predictor of postoperative AE among SUV parameters. Although SUV parameters such as SUV_max_ and SUV_mean_ are useful in assessing disease activity, they can be affected by multiple factors such as individual variations [27] and distribution of air component without SUV activity [29]. Other studies also suggested that adjusted SUV parameters have higher correlation with disease severity [17] and prognosis [18] compared to the SUV_max_ and SUV_mean_, similar to our results.

This study has some limitations. First, this was a retrospective observational study in a single center. However, the baseline characteristics of patients were similar to those in previous reports [6, 32, 33]. Secondly, PET/CT images were acquired from various PET/CT scanners. Thus, our analysis was also conducted using adjusted SUV parameters, such as the SUVR, which adjusts each individual’s ^18^F-FDG uptake [28]. Third, most subjects had malignant lung nodules. This may have affected SUV measurement in the fibrotic area. However, we attempted to minimize these effects by excluding patients with clinical findings that could affect the results (e.g. lung mass, and multiple lung nodules) and measured SUV of fibrosis area except a nodule. Lastly, some known risk factors for AE, such as treatment (home oxygen, antifibrotic agents, or steroids) before thoracic surgery or the time of surgery, were not addressed in this study, and this might affect the results. However, we could not include home oxygen and antifibrotic use in our analysis, because all patients did not use them due to the relatively preserved lung function or limited access (in South Korea, pirfenidone was covered by insurance after 2016, and most patients in this study underwent surgery before 2016). Also, most of the patients except for one did not use steroids before surgery (only one patient used steroids for a short time before surgery), and data on operation time were not available.

## Conclusion

In conclusion, our results suggest that SUV parameters may be useful in predicting postoperative AE in IPF patients. Among them, SUVR_TF_ was the best parameter and postoperative AE was more frequent in patients with high SUVR_TF_ than those with low SUVR_TF_. These findings suggest that PET/CT could provide additional information on postoperative AE in IPF patients before surgery.

## Supplementary Information

**Additional file 1: Table S1.** Comparison of postoperative complications according to the type of surgery. **Table S2.** Comparison of baseline characteristics between IPF patients with and without acute exacerbation. **Table S3.** Risk factors for postoperative complications in IPF patients assessed using univariate logistic regression analysis. **Figure S1.** Measurement of the standardized uptake value in the fibrotic area in 18F-FDG PET/CT.

## Data Availability

Any data generated and/or analysed during the current study are available from the corresponding author on reasonable request.

## Abbreviations

6MWD: 6-Minute walk distance
6MWT: Six-minute walk test
^18^F-FDG: ^18^F-fluorodeoxyglucose
PET: Positron emission tomography
AE: Acute exacerbation
ATS: American Thoracic Society
AUC: Area under the curve
CI: Confidence interval
CPI: Composite physiologic index
CRP: C-reactive protein
DLco: Diffusing capacity for carbon monoxide
FVC: Forced vital capacity
Glut-1: Glucose transporter-1
ILD: Interstitial lung disease
IPF: Idiopathic pulmonary fibrosis
KL-6: Sialylated carbohydrate antigen, Krebs von den Lungen-6
LDH: Lactate dehydrogenase
OR: Odds ratio
ROC: Receiver operating characteristics
SpO_2_: Saturation of oxygen
SP-D: Surfactant protein-D
SUV_max_: Maximum standardized uptake value
SUV_mean_: Mean standardized uptake value
SUV_meanTF_: Tissue fraction-corrected mean standardized uptake value
SUVR: Standardized uptake value ratio
SUVR_TF_: Tissue fraction-corrected mean standardized uptake value ratio
TLC: Total lung capacity
