# Supplementary material for: 18F-FDG PET/CT predicts acute exacerbation in idiopathic pulmonary fibrosis after thoracic surgery
Source: BMC Pulm Med. 2021 Sep 16;21:294. doi: 10.1186/s12890-021-01659-4 (PMC8447514; doi:10.1186/s12890-021-01659-4)
Supplement: Supplementary file 1 — Additional file 1: Table S1. Comparison of postoperative complications according to the type of surgery. Table S2. Comparison of baseline characteristics between IPF patients with and without acute exacerbation. Table S3. Risk factors for postoperative complications in IPF patients assessed using univariate logistic regression analysis. Figure S1. Measurement of the standardized uptake value in the fibrotic area in 18F-FDG PET/CT. [file 12890_2021_1659_MOESM1_ESM.docx]

**^18^F-FDG PET/CT Predicts Acute Exacerbation in Idiopathic Pulmonary Fibrosis After Thoracic Surgery**

Hee-Young Yoon, Suk Hyun Lee, Sejin Ha, Jin-Sook Ryu, Jin Woo Song

**Table S1**. Comparison of postoperative complications according to the type of surgery

| Characteristics | Total | Lobectomy | Others^†^ | P-value |
| --- | --- | --- | --- | --- |
| Numbers | 48 | 27 | 21 |  |
| Total | 21 (43.8) | 14 (51.9) | 7 (33.3) | 0.199 |
| Prolonged air leakage | 14 (29.2) | 10 (37.0) | 4 (19.0) | 0.174 |
| Acute exacerbation | 6 (12.5) | 5 (18.5) | 1 (4.8) | 0.211 |
| Others* | 4 (8.3) | 2 (7.4) | 2 (9.5) | 1.000 |
| Death | 7 (14.6) | 5 (18.5) | 2 (9.5) | 0.445 |

Data are presented as number (%), unless otherwise indicated.

* included heart failure aggravation (2.1%), delayed extubation (2.1%), myocardial infarction (2.1%), and pneumonia (2.1%).

† included wedge resection (n = 17) and segmentectomy (n = 4).

**Table S2.** Comparison of baseline characteristics between IPF patients with and without acute exacerbation

| Characteristics | AE | No-AE | P-value |
| --- | --- | --- | --- |
| Numbers | 6 | 42 |  |
| Age, years | 71.2 ± 6.6 | 67.3 ± 7.1 | 0.217 |
| Male | 6 (100.0) | 38 (90.5) | 1.000 |
| Ever-smokers | 6 (100.0) | 39 (92.9) | 1.000 |
| FVC, % predicted | 81.0 ± 16.6 | 82.2 ± 12.3 | 0.832 |
| DLco, % predicted | 74.5 ± 13.3 | 63.2 ± 18.3 | 0.154 |
| TLC, % predicted | 77.2 ± 15.1 | 79.4 ± 10.0 | 0.639 |
| 6MWD, meter | 501.3 ± 48.6 | 450.6 ± 104.6 | 0.205 |
| 6MWT, resting SpO_2_, % | 95.8 ± 2.1 | 96.7 ± 1.1 | 0.366. |
| 6MWT, lowest SpO_2_, % | 90.7 ± 6.1 | 91.4 ± 4.9 | 0.758 |
| GAP index | 2.8 ± 0.4 | 3.1 ± 1.3 | 0.591 |
| CCI | 5.0 ± 1.1 | 4.8 ± 1.5 | 0.771 |
| SUV parameters |  |  |  |
| SUV_max_ | 2.5 ± 0.8 | 2.0 ± 0.5 | 0.047 |
| SUV_mean_ | 2.0 ± 0.7 | 1.7 ± 0.4 | 0.063 |
| SUVR | 1.2 ± 0.4 | 0.9 ± 0.2 | 0.013 |
| SUV_meanTF_ | 3.1 ± 0.8 | 2.4 ± 0.6 | 0.026 |
| SUVR_TF_ | 1.8 ± 0.5 | 1.3 ± 0.4 | 0.005 |

Data are presented as mean ± SD or number (%), unless otherwise indicated.

IPF, idiopathic pulmonary fibrosis; AE, acute exacerbation; CRP, C-reactive protein; FVC, forced vital capacity; DLco, diffusing capacity for carbon monoxide; TLC, total lung capacity; 6MWD, six-minute walk distance; 6MWT, six-minute walk test; SpO2, peripheral saturation of oxygen; GAP, gender, age, and physiology; CCI, Charlson comorbidity index; SUV, standardized uptake value; SUV_max_, maximum standardized uptake value; SUV_mean_, mean standardized uptake value; SUVR, standardized uptake value ratio; SUV_meanTF_, tissue fraction-corrected mean standardized uptake; SUVR_TF_, tissue fraction-corrected standardized uptake value ratio.

**Table S3**. Risk factors for postoperative complications in IPF patients assessed using univariate logistic regression analysis

| Characteristics | Odds ratio (95% confidence interval) | P-value |
| --- | --- | --- |
| Age | 1.125 (1.012 - 1.250) | 0.029 |
| FVC | 1.008 (0.963 - 1.055) | 0.726 |
| DLco | 1.003 (0.972 - 1.036) | 0.834 |
| TLC | 1.010 (0.956 - 1.068) | 0.712 |
| 6MWD | 1.003 (0.996 - 1.009) | 0.396 |
| 6MWT, resting SpO_2_ | 0.690 (0.417 - 1.141) | 0.148 |
| 6MWT, lowest SpO_2_ | 0.938 (0.832 - 1.059) | 0.303 |
| GAP index | 1.298 (0.790 - 2.130) | 0.303 |
| CCI | 1.453 (0.942-2.284) | 0.105 |
| SUV_max_ | 2.267 (0.775 - 0.634) | 0.135 |
| SUV_mean_ | 2.846 (0.692 - 11.713) | 0.147 |
| SUVR | 3.785 (0.433 - 33.075) | 0.229 |
| SUV_meanTF_ | 3.785 (0.443 - 33.075) | 0.229 |
| SUVR_TF_ | 3.501 (0.783 - 15.647) | 0.101 |

IPF, idiopathic pulmonary fibrosis; FVC, forced vital capacity; DLco, diffusing capacity of the lung for carbon monoxide; TLC, total lung capacity; 6MWD, 6-minute walk distance; 6MWT, six-minute walk test; SpO_2_, saturation of oxygen, GAP: gender, age, and physiology; CCI, Charlson comorbidity index; SUV, standardized uptake value; SUV_max_, maximum standardized uptake value; SUV_mean_, mean standardized uptake value; SUVR, standardized uptake value ratio; SUV_meanTF_, tissue fraction-corrected mean standardized uptake; SUVR_TF_, tissue fraction-corrected standardized.

**Figure S1.** Measurement of the standardized uptake value in the fibrotic area in ^18^F-FDG PET/CT.


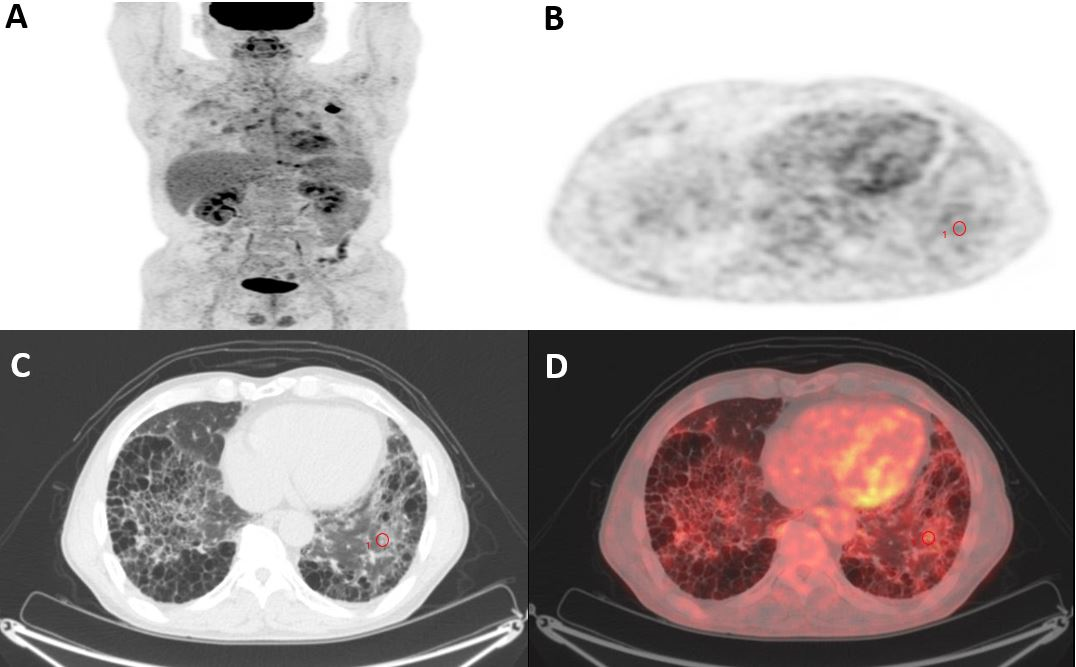


A. A coronal positron emission tomography (PET) image. B. A horizontal PET image. C. A computed tomography (CT) image. D. A combined PET and CT fusion image.

The red circles indicate the 1 cm diameter area centered on the highest ^18^F-FDG uptake in fibrotic areas. Both the maximum standardized uptake value (SUV_max_) and mean standardized uptake value (SUV_mean_) were measured in this circle.

^18^F-FDG, ^18^F-fluorodeoxyglucose; PET/CT, positron emission tomography with chest tomography.
